# Characterizing the Uncultivated Microbial Minority: towards Understanding the Roles of the Rare Biosphere in Microbial Communities

**DOI:** 10.1128/mSystems.00773-21

**Published:** 2021-08-24

**Authors:** Jimmy H. W. Saw

**Affiliations:** a Department of Biological Sciences, The George Washington University, Washington, DC, USA

**Keywords:** rare biosphere, microbial, diversity, ecology, evolution, microbial ecology, microbial evolution

## Abstract

Microbial communities are frequently numerically dominated by just a few species. Often, the long “tail” of the rank-abundance plots of microbial communities constitutes the so-called “rare biosphere,” microorganisms that are highly diverse but are typically found in low abundance in these communities. Their presence in microbial communities has only recently become apparent with advances in high-throughput sequencing technologies. Despite their low numbers, they are thought to play important roles in their communities and may function as potential members to keep the communities intact and resilient. Their phylogenetic diversity also means that they are important subjects for better understanding the interplay between microbial diversity and evolution. I propose that more efforts should be put into characterizing these poorly understood and mostly unknown microbial lineages that hold vast potentials for our understanding of microbial diversity, ecology, and evolution of life on this planet.

## COMMENTARY

The rare microbial biosphere refers to the microorganisms that are genetically diverse but are typically found in low abundance in various microbial communities (1). Despite their low abundance, they frequently constitute a phylogenetically diverse pool of microbes from all three domains of life. The rare biosphere that persists in the environment may act as seed banks of microbial diversity, allowing them to thrive when conditions are right (1, 2). Conditionally rare taxa may remain in low numbers until optimal conditions for them arise and they increase in numbers (2). Critical reviews of the rare microbial biosphere and their importance have been published previously (3, 4), and this commentary is meant to further highlight the importance of these microbes to advance the fields of microbial diversity, ecology, and evolution.

**FIG 1:**
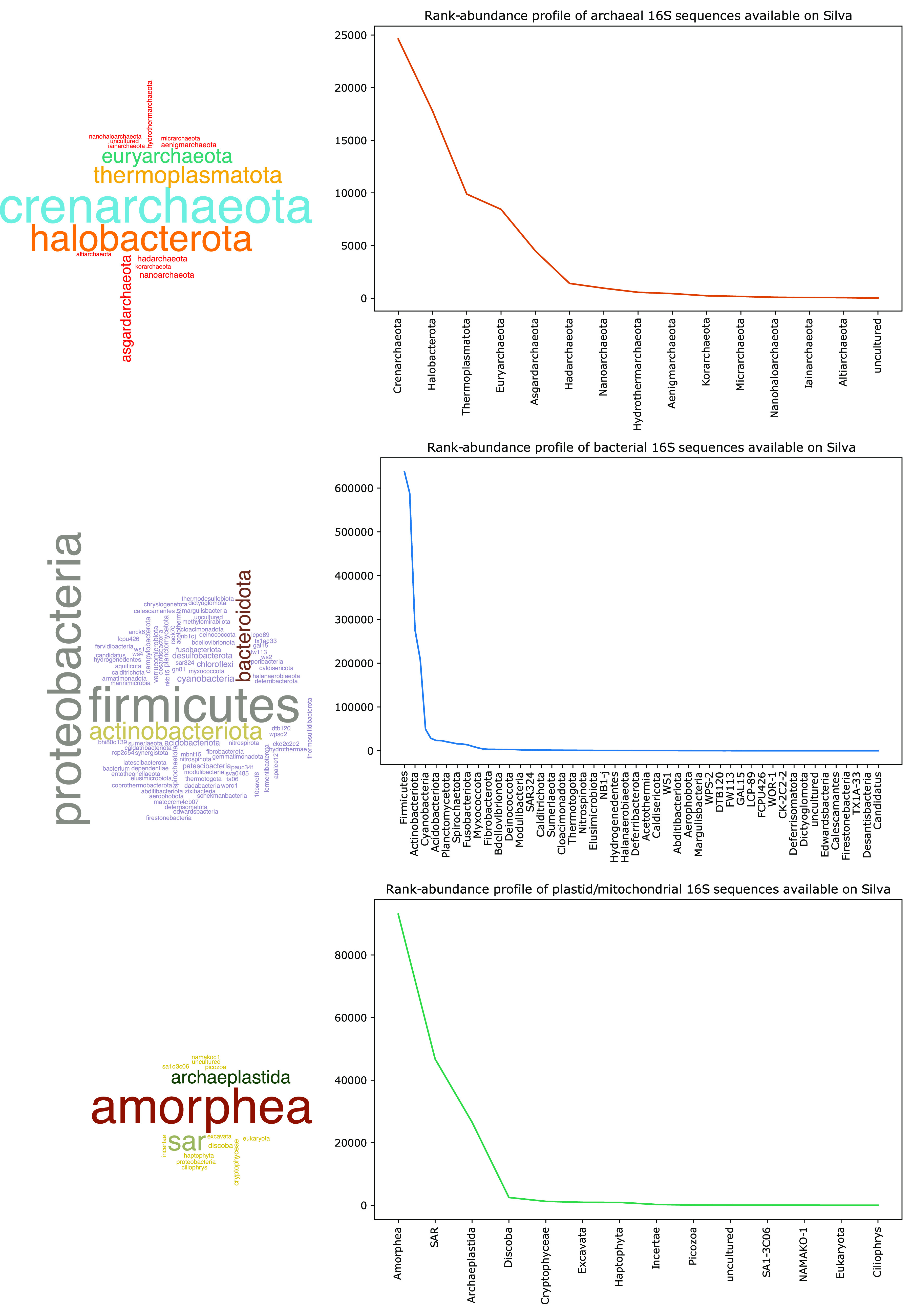
Word cloud and rank-abundance plots of archaeal, bacterial, and eukaryal phyla identified in the Silva database release 138.1. Word clouds were created from taxonomic classification of the nonredundant 16S rRNA sequences obtained from the Silva database. Note that only one out of every two bacterial phyla is shown in the figure.

A quick inspection of the taxonomic classification of the small subunit rRNA (SSU rRNA) also known as the 16S rRNA of nonredundant *Archaea*, *Bacteria*, and *Eukarya* from the Silva database (5) reveals a disparity in taxonomic representation. Most of the bacterial 16S sequences are from just a few phyla such as *Proteobacteria*, *Firmicutes*, *Actinobacteria*, and *Bacteroidetes* (Fig. 1) (Note that there are some differences between Silva and NCBI taxonomies.) Similarly, archaeal 16S rRNA sequences are mostly from *Crenarchaeota* and *Euryarchaeota* (Fig. 1). Mitochondrial and plastid sequences from eukaryotes are also unevenly distributed. As seen in Fig. 1, the long “tails” of these rank-abundance plots reveal a vast diversity of low-abundant organisms from various habitats. The Silva database typically contains longer, near-full-length 16S rRNA sequences obtained by Sanger sequencing. More taxonomically diverse lineages are likely hidden in microbial community surveys using short-read high-throughput Illumina sequencing, but they are likely discarded as noise (4).

**FIG 2:**
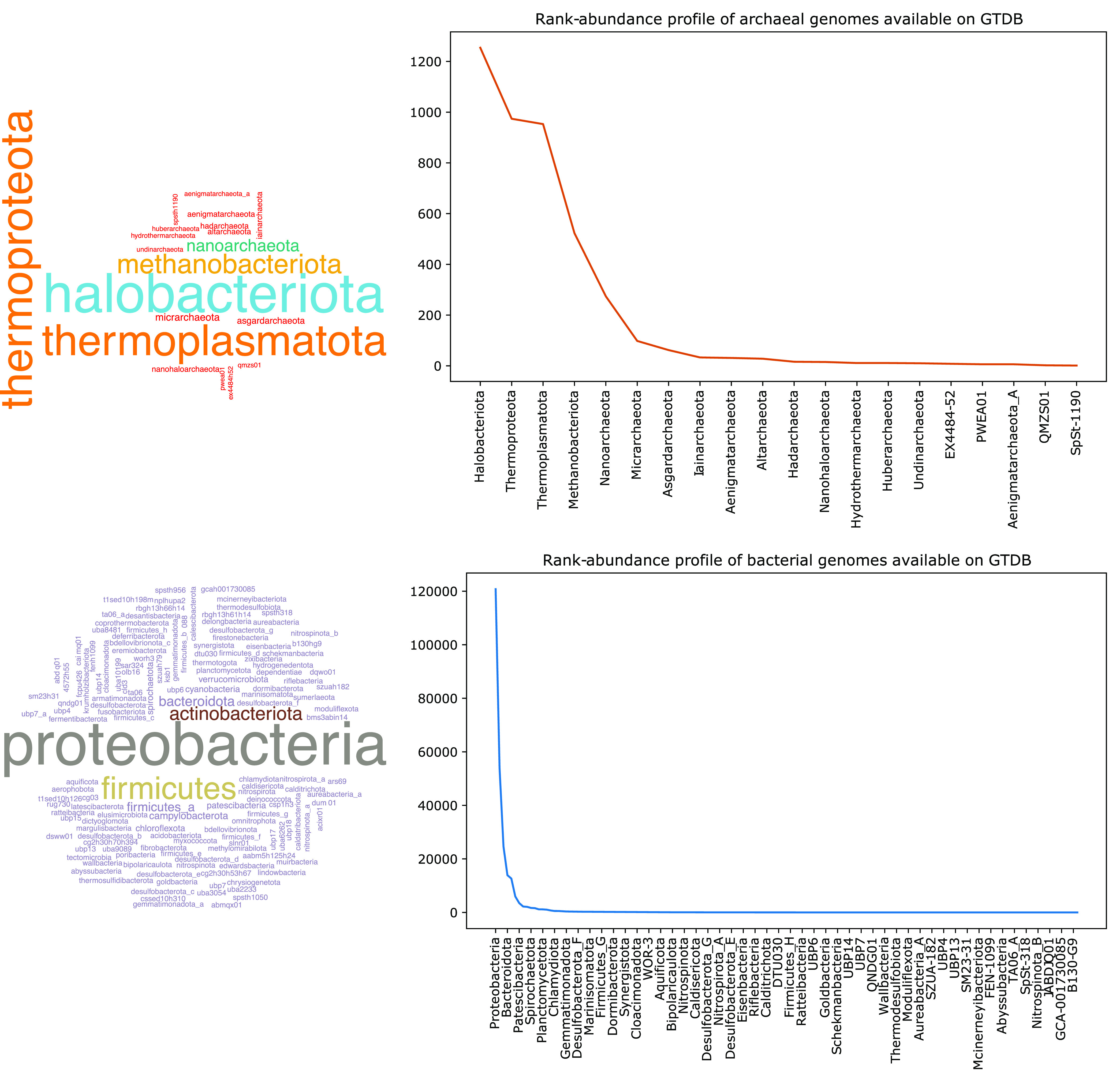
Word cloud and rank-abundance plots of archaeal and bacterial phyla identified in the Genome Taxonomy Database (GTDB). Word clouds were created from GTDB taxonomic classification of curated genomes used in the database. Note that only one out of every three bacterial phyla is shown in the figure.

Figure 2 illustrates rank-abundance and word cloud plots of taxonomic classification of a set of curated archaeal and bacterial genomes from the Genome Taxonomy Database (GTDB) (6). As seen in this figure, a vast majority of the genomes are from just a small number of archaeal and bacterial phyla: *Halobacteriota*, *Thermoproteota*, *Thermoplasmatota*, *Methanobacteriota*, *Proteobacteria*, etc.

## A FEW EXAMPLES OF RARE TAXA AND THEIR IMPORTANCE

Here, I will highlight some of the microbial lineages that I consider to belong to the rare biosphere and are important for a number of reasons.

*Odinarchaeota* are one of the few thermophilic members of the Asgard archaea, and they have thus far been recovered only from hot springs in two very remote locations: Yellowstone National Park in the United States and Taupo Volcanic Zone in New Zealand (7). Only two metagenome-assembled genomes have been constructed so far in published peer-reviewed studies. Despite being geographically separated by thousands of miles, the first-described metagenome-assembled genomes (MAGs) of *Odinarchaeota* from the United States and New Zealand encode bona fide copies of tubulin and have the smallest genome sizes among the Asgard archaea (7). They are present in only a small fraction of their respective communities based on 16S rRNA gene abundance estimates and metagenomic read recovery (7). More genomes of the *Odinarchaeota* are needed to better understand the Asgard archaeal evolution and to answer important questions on the origins of eukaryotes.

*Aigarchaeota* represent the letter A of the TACK superphylum (8), and “*Candidatus* Caldiarchaeum subterraneum” at one time was the sole member of this enigmatic archaeal phylum. It was first identified in a deep subsurface gold mine in South Africa but subsequently found in several geothermal habitats through metagenomics (9). These archaea are present in very low abundance in hot spring sediments and geothermal habitats. Similar to the *Aigarchaeota*, *Korarchaeota* represent the letter K of the TACK superphylum, and “*Candidatus* Korarchaeum cryptofilum” used to represent the sole member of the phylum for more than a decade (10). Again, thanks to metagenomics, additional members of the *Korarchaeota* are being discovered (11). Due to the position it occupies within the archaeal tree of life, thermophilic lifestyle, and lack of representation, the use of the sole member of this archaeal group tends to introduce phylogenetic artifacts that may result in wrong topologies of phylogenomic trees constructed (12). Most of the problems stem from codon biases present in the thermophilic members of these archaeal groups (13). Therefore, it is very important to obtain additional members of these enigmatic archaeal groups to improve taxonomic representation and to facilitate construction of more accurate phylogenetic trees.

Cyanobacteria of the genus *Gloeobacter* are important and may represent one of the conditionally rare taxa. First of all, they are depauperate, and only two species have been identified: one from a limestone rock in Switzerland (14) and another from a volcanic cave in Hawaii (15). Interestingly, they are numerically dominant in biofilm communities found near steam vents of Hawaii (unpublished data) and may be considered conditionally rare microbes. It has been suggested that they are common rock-dwelling cyanobacteria (16), but very few genomes of *Gloeobacter* and related deeply branching cyanobacteria currently exist in databases. There are only two cultivated species of *Gloeobacter* and a cultivated species of a sister group of *Gloeobacter* known as *Anthocerotibacter* isolated from Panama (17). A few metagenome-assembled genomes of related species were identified from Lake Vanda in Antarctica (18). These cyanobacteria occupy the deepest nodes within the cyanobacterial tree of life and are thought to be descendants of the cyanobacteria that first innovated oxygenic photosynthesis and are key to understanding how oxygenic photosynthesis evolved.

Another important lineage is a group of cyanobacteria known as *Vampirovibrionia* (formerly *Melainabacteria*) that lack the essential genes needed to perform photosynthesis (19, 20). Only a few representative genomes have been obtained from a few locations, and none of them have been isolated or cultivated yet. These rare taxa also are important to understanding the origin and evolution of oxygenic photosynthesis. Besides these examples, additional phylum-level novel lineages exist, such as WPS-2/*Eremiobacterota*, which may be important to understand the evolution of anoxygenic phototrophy (21), and GAL15 and F*ervidibacteria*, which are poorly known due to lack of representative genomes in databases but shown to be metabolically active in hot springs (22).

Here, I note an example of why increased recovery of the genomes of the rare biosphere is important. Previously known as the SAGMEG (South Africa Gold Mine Euryarchaeotic Group) (23) and later classified as a novel class, *Hadesarchaea*, based on metagenomic information (24), these archaea have been reclassified into a new phylum of their own, *Hadarchaeota*, due to increased recovery of their genomes from environmental samples (25). Phylogenomic trees constructed can only be as good as the taxa included in the tree inference, and lack of representation can mean a world of difference to the correct outcome of these studies. Therefore, an expanded genomic information of these rare taxa is very important to accurately construct phylogenetic or phylogenomic trees to understand microbial evolution.

## HOW DO WE INCREASE THE TAXONOMIC DIVERSITY OF THE RARE BIOSPHERE?

As I have highlighted in Fig. 1 and 2, the vast majority of 16S rRNA sequences and draft or complete genomes of *Archaea* and *Bacteria* belong to just a few dominant phyla. These phyla are also typically overrepresented in most microbial communities, and it is not surprising that they make up the major part of these sequence repositories.

Therefore, it is imperative that we obtain more genomic information on the vastly underrepresented but phylogenetically diverse lineages from various habitats. I propose a few approaches to increase the recovery of the genomes of these rare taxa:

1. Targeted genomic and metagenomic sequencing and exploration of the rare biosphere.
2. Targeted enrichment and cultivation of the rare biosphere based on genomic information.
3. Fluorescent-activated cell sorting (FACS) or similar approaches to enrich and sequence the rare biosphere.
4. *In situ* or mesocosm experiments to understand their physiological roles in the environment.

Targeted metagenomic sequencing of environmental samples will be crucial to recover these rare taxa and to obtain more representative genomes from various habitats. From high-throughput Illumina amplicon sequences deposited to the Sequence Read Archive (SRA) database, one can identify rare microbial lineages and identify the samples or locations they came from. Most of these studies are 16S rRNA-based surveys and do not have accompanying metagenomic data. Contacting the original authors of these studies to obtain DNA or samples to perform targeted deep sequencing of these samples would be a good start to explore and recover genomic information of these rare taxa without indiscriminately sequencing everything in sight.

Enrichment and cultivation of microbes of interest are currently having a resurgence in microbial ecology and physiology studies. More concerted cultivation efforts combined with physiological experiments will be needed to characterize these poorly represented and understood microbes and their roles in various habitats. However, cultivation of previously uncultivated microbes is still a tremendous challenge, and more efforts will need to be put in to obtain pure cultures. Various approaches to cultivate previously uncultivated microbes have been reported, and they are promising methods to target isolation of the rare biosphere (26, 27). Single-cell genomics is one of the solutions to the problem with cultivating the rare biosphere, but it can be quite expensive and single-cell amplified genomes (SAGs) tend to be highly incomplete. Because we are targeting the rare biosphere that tends to be found in low abundance, chances of sorting them out of the pool of abundant but less interesting microbes are very limited.

One solution might be to use methods such as FACS to size-fractionate microbial cells and sequence minimetagenomes to exclude more abundant lineages in the metagenomic sequences. If one can flow-sort a microbial population by size and other properties, it might be possible to reduce the number of dominant taxa from the sequencing pool. There is still a chance that the dominant and the rare taxa may have similar cell sizes in certain communities, but if we couple it with fluorescent tags bound to cells of interest, the method might be feasible. Lastly, we should not forget that physiological experiments are important to understanding what the rare biosphere does in its natural environment. Polyphasic approaches using a combination of targeted sequencing, isolation of target species, and physiological experiments will help us understand more about the roles of the rare biosphere in microbial communities.

### Data availability.

Data and code used to create Fig. 1 and 2 can be accessed at https://github.com/SawLabGW/mSystems_ECSC2021.
